# Modifiable dementia risk factors and AT(N) biomarkers: findings from the EPAD cohort

**DOI:** 10.3389/fnagi.2024.1346214

**Published:** 2024-02-07

**Authors:** Eddy Roccati, Aidan David Bindoff, Jessica Marie Collins, Joshua Eastgate, Jay Borchard, Jane Alty, Anna Elizabeth King, James Clement Vickers, Margherita Carboni, Chad Logan, EPAD Consortium

**Affiliations:** ^1^Wicking Dementia Research and Education Centre, University of Tasmania, Hobart, TAS, Australia; ^2^Royal Hobart Hospital, Hobart, TAS, Australia; ^3^Roche Diagnostics International Ltd, Rotkreuz, Switzerland; ^4^Roche Diagnostics GmbH, Penzberg, Germany; ^5^Department of Radiology and Nuclear Medicine, University of Amsterdam, De Boelelaan, Amsterdam, Netherlands

**Keywords:** Alzheimer’s disease, amyloid-beta, tau, neurodegeneration, modifiable dementia risk, biomarkers

## Abstract

**Introduction:**

Modifiable risk factors account for a substantial proportion of Alzheimer’s disease (AD) cases and we currently have a discrete AT(N) biomarker profile for AD biomarkers: amyloid (A), p-tau (T), and neurodegeneration (N). Here, we investigated how modifiable risk factors relate to the three hallmark AT(N) biomarkers of AD.

**Methods:**

Participants from the European Prevention of Alzheimer’s Dementia (EPAD) study underwent clinical assessments, brain magnetic resonance imaging, and cerebrospinal fluid collection and analysis. Generalized additive models (GAMs) with penalized regression splines were modeled in the AD Workbench on the NTKApp.

**Results:**

A total of 1,434 participants were included (56% women, 39% *APOE* ε4+) with an average age of 65.5 (± 7.2) years. We found that modifiable risk factors of less education (*t* = 3.9, *p* < 0.001), less exercise (*t* = 2.1, *p* = 0.034), traumatic brain injury (*t* = −2.1, *p* = 0.036), and higher body mass index (*t* = −4.5, *p* < 0.001) were all significantly associated with higher AD biomarker burden.

**Discussion:**

This cross-sectional study provides further support for modifiable risk factors displaying neuroprotective associations with the characteristic AT(N) biomarkers of AD.

## Introduction

Dementia remains one of the greatest global health, social, and economic challenges of the 21st century (Livingston et al., 2017), yet evidence suggests up to 40% of dementia risk is modifiable (Livingston et al., 2020). Modifiable risk factors for dementia are education, hearing loss, traumatic brain injury (TBI), hypertension, alcohol consumption, obesity, smoking, depression, social isolation, physical inactivity, air pollution, and diabetes (Livingston et al., 2020). With research indicating that many dementia cases could be avoided by changing lifestyle behaviors (Livingston et al., 2020), addressing modifiable risk factors could prevent or delay over 40 million cases worldwide by 2050 (Moore et al., 2015). To our knowledge, modifiable risk factors contribute a significant proportion to dementia risk (Livingston et al., 2020), are highly prevalent in the community (Yaffe, 2018), and are exacerbated by low socioeconomic status (Kezios et al., 2022); however, modifiable risk factors are often overlooked as potential therapeutic targets for risk reduction.

Targeting modifiable risk factors entails a suite of positive impacts not only on dementia but also on the prevention of other chronic conditions such as cardiovascular disease and cancer, which share similar risk factors (Edwards et al., 2019). Intervening earlier in the disease course is likely to bring about greater benefits for individuals, where effective interventions for blood pressure reduction, smoking cessation, and prevention of diabetes deliver the greatest efficacy in significantly reducing health care expenditure, reducing dementia prevalence, and producing quality-adjusted life-year gains (Mukadam et al., 2020). There is also evidence for clustering incremental risk from modifiable risk factors. Modifiable risk factors tend to co-occur (Griffin et al., 2014; Morris et al., 2016), where clusters typically consist of smoking, excess alcohol consumption, and physical inactivity (Peters et al., 2019).

Alzheimer’s disease (AD) has a prolonged period of ‘silent’ degeneration, a preclinical phase that lasts up to decades prior to clinical symptom onset (Sperling et al., 2011). During this phase, pathophysiological processes of beta-amyloid (Aβ) accumulation, phosphorylated-tau (p-tau) aggregation, and neurodegeneration emerge, with subtle cognitive deficits developing alongside (Sperling et al., 2011). Under this framework, the National Institute on Aging—Alzheimer’s Association (NIA-AA) has established discrete biomarker profiles to discriminate Alzheimer’s pathological change from non-Alzheimer’s pathological change (Jack et al., 2018). The NIA-AA’s AT(N) profiling system classifies individuals based on the presence of one or more hallmark biomarkers of AD: amyloid β (A), p-tau (T), and neurodegeneration (N). This window of biologically burdened, yet cognitively unimpaired, pathological change presents an ideal opportunity to intervene with modifiable risk factors that can impact dementia risk. By the time clinical symptoms emerge, the underlying AD pathology has likely entered an advanced stage, limiting the impact of interventions (Reiman et al., 2011).

We have strong evidence from around the world that targeting modifiable risk factors can maintain and improve cognitive function (Ngandu et al., 2015), as well as reduce the risk for AD and other forms of dementia (Kivipelto et al., 2018), which is evidenced by findings from a systematic review (Coley et al., 2022) recommending age-, sex-, and factor-specific lifestyle modifications. Whether an intervention targets physical activity and hypertension or is multidomain in nature, risk factor reduction efforts for dementia will likely overlap with preventative efforts for other chronic conditions. This aligns with a global effort to improve the quality of life throughout aging, accounting for the shared pathways and biological mechanisms of age-related chronic conditions. Furthermore, recent advancements have come alongside calls for biomarker results to be disclosed to research participants (Grill and Karlawish, 2022). We currently have the tools to safely disclose biomarker insights in order to foster positive lifestyle modification and create supportive environments for individuals to reduce their risk (Frisoni et al., 2023).

Despite strong evidence for a biologically defined AD continuum (Jack et al., 2018) and modifiable risk amelioration (Livingston et al., 2020), there are very few studies investigating modifiable risk categories against the biologically driven AT(N) biomarkers. Addressing this gap is important as it opens earlier periods of life to intervention. In this study, we aimed to investigate how modifiable risk factors are related to the three hallmark AD biomarkers: amyloid β (A), p-tau (T), and neurodegeneration (N). We hypothesized that we would observe associations between adherence to modifiable risk factors and increased AD biomarker burden measured via AT(N) criteria and that these associations would be of use to clinicians, researchers, and caregivers in assessing the biological and modifiable risk profile of individuals.

## Materials and methods

### Participants

Participants were drawn from the European Prevention of Alzheimer’s Dementia (EPAD) Longitudinal Cohort Study (LCS) (Ritchie et al., 2020). A full protocol has been published previously (Solomon et al., 2018). Briefly, EPAD is a prospective, multicenter, pan-European longitudinal cohort study. Participants were recruited across 21 different European sites and were eligible if they were at least 50 years of age, had completed at least 5 years of formal education, and did not have a dementia diagnosis at baseline. Participants underwent clinical and neurological assessments including a mini-mental state examination (MMSE), brain magnetic resonance imaging (MRI), lumbar puncture for cerebrospinal fluid (CSF), and comprehensive neuropsychological assessment. For the purposes of this study, we used only EPAD LCS Visit 1 (V1) baseline data (EPAD LCS-v.IMI V1 [*n* = 2,737, 55.8% women]) as we intended to investigate the clinical utility of a biomarker + modifiable risk factor panel to foster early detection and intervention in healthy populations. For the neurodegeneration analysis, there was a small fraction (*n* = 17, 1.3%) of participants returning extremely low values for L and R hippocampal volume; therefore, we excluded those participants with a value of less than 1,000 mm^3^ for total hippocampal volume (THV). Hearing loss was removed from all models due to missing data and insufficient levels for GAM comparison. We did not apply any other exclusion criteria to the study population. Baseline data (V1) from 1,474 participants were included, with sample size numbers varying according to the availability of neuroimaging and lumbar puncture data. The study was approved by the ethical committees of all participating EPAD centers. All study participants provided written informed consent prior to the collection of any study data. All procedures were conducted in accordance with the Declaration of Helsinki.

### Non-modifiable risk factors

Non-modifiable criteria consisted of age, sex, and presence of the apolipoprotein epsilon 4 allele (*APOE* ε4). Age at baseline (in years) was calculated from age (years) and age (months) reported at the time of assessment. Sex at birth was also collected in demographic assessments. *APOE* genetic analysis was carried out on blood samples collected during baseline assessments. *APOE* allelic carriage was stratified by two ε alleles for six different combinations: ε2/ε2, ε2/ε3, ε3/ε3, ε3/ε4, ε4/ ε4, and ε4/ε2. We also stratified based on ε4 presence as ε4+ (ε3/ε4, ε4/ε4, or ε4/ε2) and ε4- (ε2/ε2, ε2/ε3, or ε3/ε3).

### Modifiable risk factors

Participants completed a variety of demographic, clinical, medical history, and lifestyle surveys at EPAD assessments. Survey collection was standardized across all European sites to ensure consistency of measurement. Data collection protocols were harmonized and were in accordance with the International Conference on Harmonization (ICH). Ten modifiable risk criteria from Livingston et al.’s *Lancet Commission* were collected (Livingston et al., 2020). We were unable to account for social isolation and air pollution due to data availability. Education (in years) data were collected in demographics. Medical questionnaires asked about the history of diabetes (type 1 or 2), obesity, hypertension, TBI, depression, and hearing loss. Lifestyle factors collected were smoking (never/past/current), alcohol consumption (units/week), and frequency of physical activity, defined as leisure-time physical activity that lasted at least 20 min, and caused breathlessness and sweating (daily, 2–3 times a week, once a week, 2–3 times a month, a few times a year, or not at all) (Rovio et al., 2005). We dichotomized adherence to modifiable risk factors based on existing evidence (Livingston et al., 2020) and stratified the cohort based on fulfilling the following criteria: medical history (yes for diabetes, hypertension, TBI, depression, obesity, and hearing loss); lifestyle (physical inactivity: not at all/a few times a year; smoking: past/current; and alcohol: 2–6 units per day); and education (≤12 years).

### Cerebrospinal fluid

CSF was obtained at baseline assessment using a pre-analytical protocol harmonized across study sites. Analyses were performed using the Roche ELECSYS^®^ immunoassays (Roche Diagnostics International Ltd., Rotkreuz, Switzerland) at the University of Gothenburg (Solomon et al., 2018). Concentrations of Aβ1-42 and p-tau 181 were determined according to the manufacturer’s instructions.

### Magnetic resonance imaging

Brain MRI scans were performed with standardized acquisition protocols. Images were centrally evaluated by experienced raters and blinded to neuropsychological and clinical data. Scans were visually assessed for white matter hyperintensities, perivascular spaces, microbleeds, medial temporal lobe atrophy (MTA), and posterior cortical atrophy. Regional measures for white matter volume, gray matter volume, and hippocampal volume were determined using a segmentation process based on atlas propagation with the Learning Embeddings for Atlas Propagation framework (Jack et al., 2017). THV is the sum of left hippocampal volume (LHV) and right hippocampal volume (RHV), all expressed in mm^3^.

### AT(N) biomarkers

AT(N) biomarker profiling and classification of EPAD participants was based on published research (Jack et al., 2018; Ebenau et al., 2020; Ingala et al., 2021). Eligible EPAD participants had their AT(N) biomarkers measured in CSF (CSF Aβ1-42 [pg/mL] and CSF p-tau 181 [pg/mL]) and MRI (THV [mm^3^]). Central laboratories conducted a harmonized protocol to ensure measurement consistency and interpretation: CSF at the University of Gothenburg, genetics at the University of Edinburgh, and neuroimaging at the Amsterdam University Medical Center (Solomon et al., 2018). Participants were classified into presence (+) or absence (−) of abnormal CSF Aβ1-42 (“A”), CSF p-tau 181 (“T”), and neurodegeneration (“N”). For A+/−, participants were split using a cut-off of CSF Aβ1-42: < 1,000 pg./mL classified as A+ and ≥ 1,000 pg./mL classified as A-. For T+/−, CSF p-tau 181: > 27 pg./mL was classified as T+ and ≤ 27 pg./mL was classified as T-. For N+/−, participants’ age and MTA average (L/R) were used: participants were classified as N+ if their age was <65 years and their MTA average (L/R) was ≥1 or their age was ≥65 years and their MTA average (L/R) was ≥1.5; all other participants were classified as N-. AT(N) criteria were further classified based on A+/−, T+/−, and N+/−, stratifying participants into 8 groups: A−/T-/N-, A−/T-/N+; A−/T+/N-, A−/T+/N+, A+/T-/N-, A+/T-/N+, A+/T+/N-, and A+/T+/N+.

### Statistical methods

Data were accessed via the NeuroToolKit (NTK) Application (NTKApp, BetaVersion, 2022) on the AD Workbench, a powerful cloud-based data-sharing platform designed by the Alzheimer’s Disease Data Initiative (ADDI). All analyses were performed using R code on the NTK’s Analysis module. Demographic characteristics were expressed as frequencies (percentage) and mean ± standard deviation (SD). *T*-test for continuous variables and chi-squared test for categorical variables were used to test the difference between men and women and discrete AT(N) groups. Generalized additive models (GAMs) with regression splines were used to model the associations between modifiable risk factor adherence and AT(N) biomarkers. Two GAMs were fit against each individual AT(N) biomarker as the dependent variable. The goodness of fit was determined by interpreting Akaike’s Information Criterion (AIC). Age (in years) and total MMSE (out of 30) were smoothed due to being non-linearly associated with AT(N) biomarkers. Since AD is a disease of aging, an appropriate adjustment of the age that acknowledges this non-linearity was made to more accurately estimate the effects of modifiable risk factors independent of age. Education (in years) and BMI (weight in kg * height in m^2^) were included in GAMs due to their better fit than binary variables (less education/obesity). Following an inspection of Q-Q plots, AT(N) biomarkers were log-transformed to approximate the normal distribution of residuals; however, untransformed values were used for visualization. Modifiable risk factor independent variables were tested against the individual AT(N) dependent variables in both individual and multiple independent variable models; however, the results were similar and thus multiple independent variables are shown in Model 1. Model 1 was adjusted for all modifiable criteria (education, TBI, hearing, hypertension, alcohol, BMI, smoking, depression, exercise, and diabetes), all unmodifiable criteria (age, sex, and *APOE* ε4 presence), and cognition (MMSE). Model 2 was adjusted for all significant variables in Model 1 together with unmodifiable covariates. In all GAMs, Model 1 (with all unmodifiable and modifiable criteria) displayed the best fit, with the lowest relative AIC. Figures were produced using the ggplot2 package for R in NTK Analysis. Reproducible NTK Analysis code and data are both available from the AD Workbench.

### Data availability

All EPAD data are available via the NTKApp on the AD Workbench, designed by ADDI. NTKApp is accessible at: https://www.alzheimersdata.org/ntk. R code is available upon request to the corresponding author, Dr. Eddy Roccati (eddy.roccati@utas.edu.au).

The EPAD Longitudinal Cohort Study brings together participants from 21 sites across Europe. For a full list of collaborators contributing to open-access data, please visit: https://ep-ad.org/open-access-data/overview/.

## Results

Baseline demographic statistics are displayed in Table 1. Participants were predominantly women (56%, *N* = 821), with an average age of 14.4 (SD 3.7) years of education and the highest genotypic prevalence for APOE being ε3 homozygotes (52%, *N* = 714). The overall penetrance for *APOE* ε4 allele was 39.0% in both women and men. Men were significantly older, more educated, and had a higher prevalence of alcohol misuse, smoking, and physical inactivity. Women demonstrated significantly higher rates of depression, higher CSF Aβ1-42, and lower THV than men. The most commonly reported risk factors were obesity (59.3%), smoking (53.9%), less education (34.4%), physical inactivity (21.5%), and alcohol consumption (12.4%). A total of 338 (22.9%) participants reported adhering to zero risk factors, 340 (23.1%) of them reported one, 440 (29.9%) of them reported two, 272 (18.5%) of them reported three, 79 (5.4%) of them reported four, and 5 (0.3%) of them reported five.

**Table 1:** Demographic statistics of included EPAD participants (*N* = 1,474).

		Women	Men	Total	*p*-value
N (%)		821 (56%)	653 (44%)	1,474	
Age at baseline in years (SD)		65.5 (7.2)	66.7 (7.4)	66.0 (7.3)	<0.001
Education in years (SD)		14.1 (3.7)	14.7 (3.8)	14.4 (3.7)	0.004
MMSE (SD)		28.4 (1.9)	28.4 (2)	28.4 (1.9)	0.809
*APOE* ε4 genotype					0.211
	e2e2	2 (0.3%)	2 (0.3%)	4 (0.3%)	
	e2e3	60 (7.8%)	57 (9.6%)	117 (8.6%)	
	e3e3	410 (53.2%)	304 (51.1%)	714 (52.3%)	
	e2e4	18 (2.3%)	17 (2.9%)	35 (2.6%)	
	e3e4	249 (32.3%)	186 (31.3%)	435 (31.9%)	
	e4e4	31 (4%)	29 (4.9%)	60 (4.4%)	
A: CSF Aβ1-42 pg/mL (SD)		1427.7 (798.9)	1325.8 (669.7)	1382.6 (745.9)	0.016
T: CSF p-tau 181 pg/mL (SD)		20.0 (11.1)	20.0 (11)	20.0 (11.1)	0.977
N: THV mm^3^ (SD)		4,591 (767.3)	4896.2 (823.9)	4724.8 (806.8)	
Less education					0.089
	No	479 (63.6%)	401 (68.2%)	880 (65.6%)	
	Yes	274 (36.4%)	187 (31.8%)	461 (34.4%)	
Self-report hearing difficulty					0.785
	No	21 (95.5%)	25 (89.3%)	46 (92%)	
	Yes	1 (4.5%)	3 (doi:10.7%)	4 (8%)	
TBI					0.533
	No	735 (98.1%)	581 (97.5%)	1,316 (97.8%)	
	Yes	14 (1.9%)	15 (2.5%)	29 (2.2%)	
Hypertension					0.057
	No	706 (94.3%)	545 (91.4%)	1,251 (93%)	
	Yes	43 (5.7%)	51 (8.6%)	94 (7%)	
Alcohol > 21 units p/w					<0.001
	No	713 (93.8%)	475 (79.7%)	1,188 (87.6%)	
	Yes	47 (6.2%)	121 (20.3%)	168 (12.4%)	
Smoking					<0.001
	No	395 (50.3%)	252 (40.9%)	647 (46.1%)	
	Yes	391 (49.7%)	364 (59.1%)	755 (53.9%)	
Obesity					0.157
	No	292 (39%)	256 (43%)	548 (40.7%)	
	Yes	457 (61%)	340 (57%)	797 (59.3%)	
Depression					0.009
	No	699 (93.3%)	576 (96.6%)	1,275 (94.8%)	
	Yes	50 (6.7%)	20 (3.4%)	70 (5.2%)	
Physical inactivity					0.021
	No	599 (76.2%)	501 (81.5%)	1,100 (78.5%)	
	Yes	187 (23.8%)	114 (18.5%)	301 (21.5%)	
Diabetes					0.914
	No	736 (98.3%)	587 (98.5%)	1,323 (98.4%)	
	Yes	13 (1.7%)	9 (1.5%)	22 (1.6%)	

All available data are displayed. All values shown are n (%) unless otherwise stated. *p*-values are provided for group comparisons (men vs. women) between either continuous (one-way ANOVA) or categorical (Chi-squared) data. Significance was set at a *p* < 0.05. SD, standard deviation; MMSE, mini-mental state examination; APOE, apolipoprotein epsilon E; Aβ, beta-amyloid; p-tau, phosphorylated tau; CSF, cerebrospinal fluid; THV, total hippocampal volume; TBI, traumatic brain injury.

### AT(N) comparison

Summary statistics stratified by amyloid classification are displayed in Supplementary Table 1. A+ participants (33%, *N* = 406) were significantly older, had higher *APOE* ε4 penetrance, higher CSF p-tau 181, lower THV, and lower MMSE scores than A- participants (67%, *N* = 810). For modifiable risk factors, A+ participants had a significantly higher prevalence of alcohol misuse and obesity. All other modifiable risk factors were non-significant.

Summary statistics stratified by tau classification are displayed in Supplementary Table 2. T+ participants (18%, *N* = 214) were significantly older, less educated, and had higher penetrance for *APOE* ε4, lower THV, and lower MMSE scores than T- participants (82%, *N* = 1,001). Compared with T–, T+ participants had significantly higher rates of smoking and obesity.

Summary statistics stratified by neurodegeneration classification are displayed in Supplementary Table 3. N+ participants (15%, *N* = 195) were significantly older and displayed higher CSF Aβ1-42 and lower MMSE scores than N- participants (85%, *N* = 1,105). In terms of modifiable risk factors, N+ participants only had a significantly higher prevalence of smoking than N- participants.

### AT(N) generalized additive models

**Figure 1:**
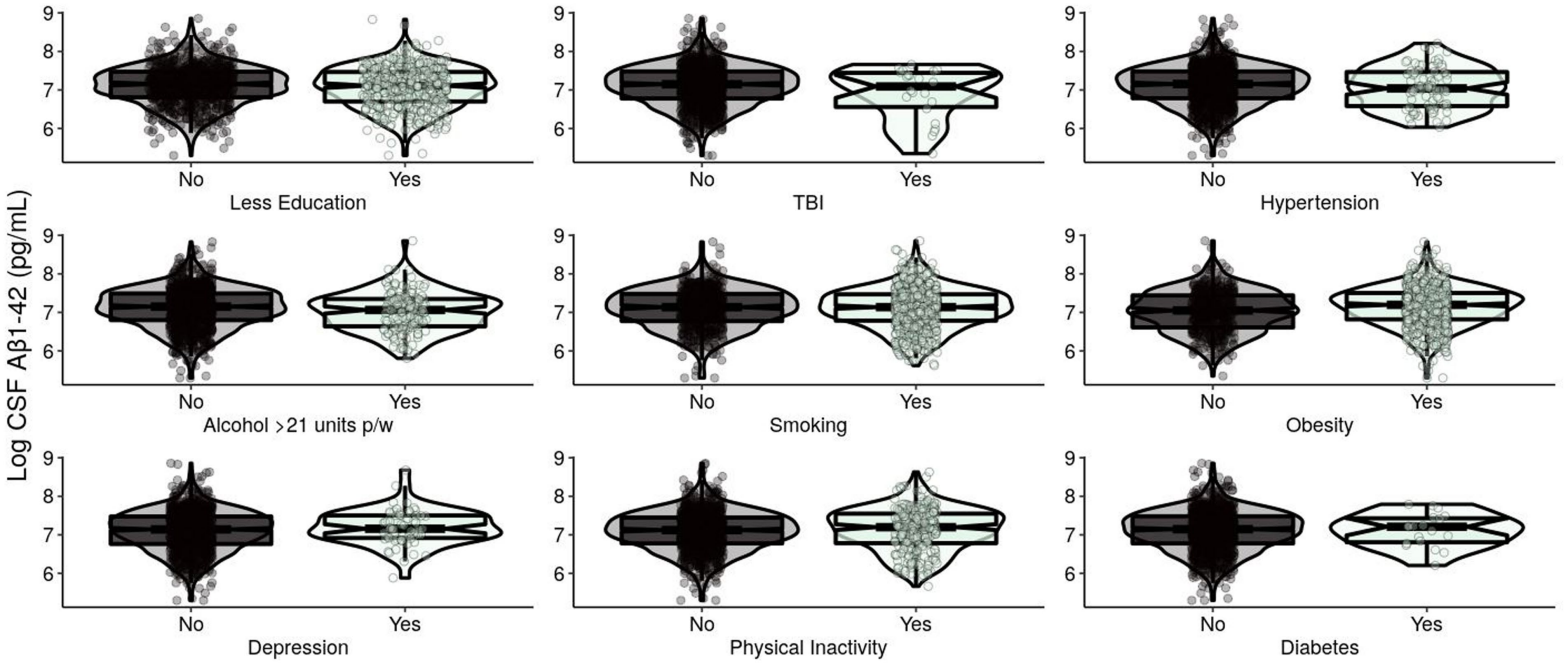
Levels of log-transformed CSF Aβ1-42 (pg/mL) of EPAD participants grouped by adherence to modifiable risk factors. Log-transformed levels of CSF beta-amyloid 1–42 (pg/mL) are presented. Raw amyloid +/− was classified using a cut-off of CSF Aβ1-42: < 1,000 pg./mL classified as A+ and ≥ 1,000 pg./mL classified as A−. CSF, cerebrospinal fluid; Aβ, beta-amyloid; EPAD, European Prevention of Alzheimer’s Dementia; TBI, traumatic brain injury.

For A in Model 1, *APOE* ε4 presence and TBI were significantly negatively associated with CSF Aβ1-42 (Table 2; Figure 1). In Model 2, these results remained significant after removing non-significant modifiable risk factors. Smoothed age and MMSE were also significantly associated with CSF Aβ1-42.

**Table 2:** Detailed summary table of generalized additive model (GAM) regression results for AT(N) individual biomarkers.

Parametric coefficients																								
	A: log CSF Aβ1-42 pg/mL								T: log CSF p-tau 181 pg/mL								N: log THV mm^3^							
	Model 1				Model 2				Model 1				Model 2				Model 1				Model 2			
	Estimate	Std Error	*t*-value	Pr (>|t|)	Estimate	Std error	*t*-value	Pr (>|t|)	Estimate	Std error	*t*-value	Pr (>|t|)	Estimate	Std error	*t*-value	Pr (>|t|)	Estimate	Std error	*t*-value	Pr (>|t|)	Estimate	Std error	*t*-value	Pr (>|t|)
Intercept	**7.206**	**0.12**	**60.21**	**<0.001**	**7.243**	**0.023**	**311.5**	**<0.001**	**3.211**	**0.095**	**33.90**	**<0.001**	**3.127**	**0.071**	**43.78**	**<0.001**	**8.353**	**0.027**	**3doi:10.7**	**<0.001**	**8.354**	**0.025**	**335.10**	**<0.001**
Sex: Men	−0.03	0.032	−1.00	0.318	−0.05	0.03	−1.5	0.133	0.003	0.025	0.100	0.920	0.007	0.024	0.279	0.780	**0.066**	**0.007**	**8.977**	**<0.001**	**0.068**	**0.007**	**doi:10.18**	**<0.001**
*APOE* ε4: Yes	**−0.27**	**0.031**	**−8.64**	**<0.001**	**−0.27**	**0.031**	**−8.61**	**<0.001**	**0.143**	**0.025**	**5.749**	**<0.001**	**0.148**	**0.024**	**6.272**	**<0.001**	−0.01	0.007	−0.880	0.380	−0.01	0.007	−1.39	0.165
Education (in years)	0.002	0.004	0.396	0.692					−0.01	0.003	−1.69	0.091					**0.003**	**0.001**	**3.301**	**<0.001**	**0.004**	**0.001**	**3.876**	**<0.001**
TBI	**−0.24**	**0.12**	**−1.97**	**0.049**	**−0.25**	**0.12**	**−2.1**	**0.036**	−0.16	0.095	−1.65	0.100					−0.01	0.027	−0.31	0.754				
Hypertension	0.048	0.062	0.776	0.438					0.007	0.05	0.14	0.889					0.001	0.014	0.085	0.932				
Alcohol > 21 units p/w	−0.05	0.049	−1.08	0.282					−0.05	0.039	−1.19	0.236					0.008	0.011	0.681	0.496				
Smoking	0.012	0.031	0.383	0.702					0.035	0.024	1.431	0.153					−0.00	0.007	−0.17	0.862				
BMI	−0.00	0.004	−0.17	0.864					**−0.01**	**0.003**	**−4.35**	**<0.001**	**−0.01**	**0.003**	**−4.54**	**<0.001**	**0.002**	**0.001**	**2.04**	**0.042**	**0.002**	**0.001**	**1.973**	**0.049**
Depression	0.103	0.065	1.572	0.116					0.035	0.052	0.672	0.502					−0.00	0.015	−0.06	0.949				
Physical inactivity	0.062	0.037	1.67	0.095					**0.058**	**0.029**	**1.989**	**0.047**	**0.06**	**0.028**	**2.121**	**0.034**	−0.00	0.008	−0.36	0.722				
Diabetes	−0.04	0.112	−0.32	0.751					−0.06	0.089	−0.69	0.491					−0.03	0.027	−0.96	0.335				

Approximate significance of smooth terms:																								
	edf	Ref.df	*F*	*p*-value	edf	Ref.df	*F*	*p*-value	edf	Ref.df	*F*	*p*-value	edf	Ref.df	*F*	*p*-value	edf	Ref.df	*F*	*p*-value	edf	Ref.df	*F*	*p*-value
s(age)	1	1	4.053	0.044	1	1.001	4.552	0.033	3.06	3.872	33	<0.001	3.536	4.446	35.74	<0.001	2.455	3.124	26.11	<0.001	2.396	3.048	33.51	<0.001
s(MMSE)	3.43	4.244	12.14	<0.001	3.353	4.155	12.57	<0.001	3.157	3.924	13.38	<0.001	1.574	1.968	30.32	<0.001	2.253	2.828	16.69	<0.001	2.372	3.011	20.16	<0.001

Generalized additive model (GAM) regression results for natural log of individual AT(N) biomarkers. Model 1 adjusted for all modifiable criteria (education [in years], TBI, hypertension, alcohol, BMI, smoking, depression, exercise, and diabetes), unmodifiable criteria (age, sex, and APOE presence), and cognition (MMSE). Model 2 adjusted for all significant variables in Model 1 together with unmodifiable covariates. Thin plate regression splines applied to smoothed terms of age and cognition (MMSE). Significance was set at a *p* < 0.05. edf, effective degrees of freedom; Ref.df, Reference degrees of freedom; MMSE, mini-mental state examination; APOE, apolipoprotein epsilon E; Aβ, beta-amyloid; p-tau, phosphorylated tau; CSF, cerebrospinal fluid; THV, total hippocampal volume; TBI, traumatic brain injury. Bold values represent significance (*p* <0.05).

**Figure 2:**
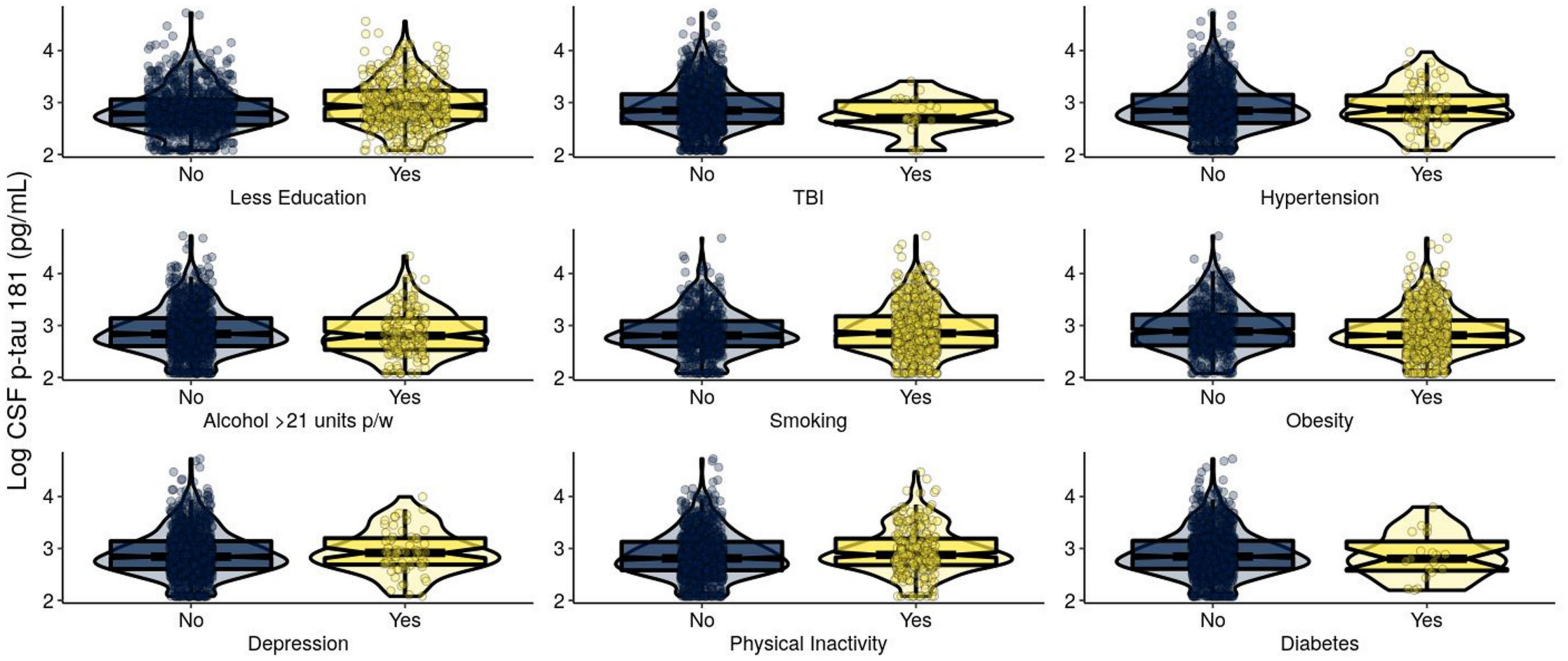
Levels of log-transformed CSF p-tau 181 (pg/mL) of EPAD participants grouped by adherence to modifiable risk factors. Log-transformed levels of CSF p-tau 181 (pg/mL) are presented. Raw tau +/− was classified using a cutoff of CSF p-tau 181: > 27 pg./mL classified as T+ and ≤ 27 pg./mL classified as T−. CSF, cerebrospinal fluid; p-tau, phosphorylated tau; EPAD, European Prevention of Alzheimer’s Dementia; TBI, traumatic brain injury.

For T in Model 1, *APOE* ε4 and BMI were significantly positively associated with CSF p-tau 181 (Table 2; Figure 2). In Model 2, these results remained significant. BMI was significantly negatively associated with CSF p-tau 181. Smoothed age and MMSE were significantly associated with CSF p-tau 181.

**Figure 3:**
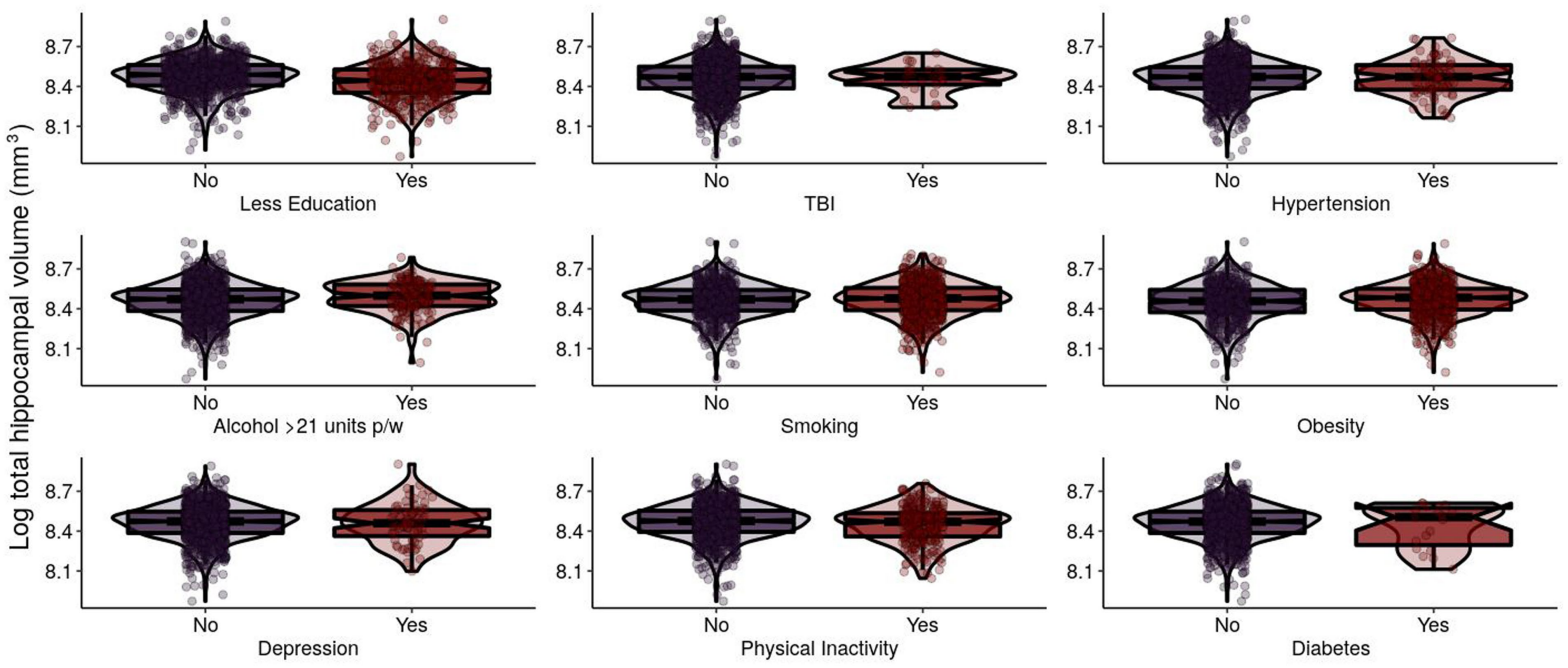
Log-transformed total hippocampal volume (mm^3^) of EPAD participants grouped by adherence to modifiable risk factors. Log-transformed levels of total hippocampal volume (mm^3^) are presented. Total hippocampal volume (THV) is the sum of left hippocampal volume (LHV) and right hippocampal volume (RHV). EPAD, European Prevention of Alzheimer’s Dementia; TBI, traumatic brain injury.

For N in Model 1, sex, education, and BMI were significantly positively associated with THV (Table 2; Figure 3). In Model 2, these results remained significant. Smoothed age and MMSE were significantly associated with THV.

## Discussion

In a large-scale community cohort of cognitively healthy participants from the EPAD study, we found significant relationships between modifiable risk factors for dementia and the hallmark AT(N) biomarkers that precede clinical symptoms of AD and other forms of dementia. Self-reported history of TBI was significantly associated with lower levels of Aβ1-42 levels in CSF; physical inactivity and lower BMI were significantly associated with higher levels of p-tau 181 in CSF; and higher education and higher BMI were both significantly associated with higher THV.

We found that the history of TBI was significantly associated with lower CSF Aβ1-42. Even after adjusting for age, sex, *APOE* ε4 presence, and cognition, the relationship remained, which is concordant with previous research into post-mortem Aβ plaque pathology following survivors of TBI (Johnson et al., 2012) and acute ventricular CSF Aβ1-42 elevations in the initial week following severe TBI (Olsson et al., 2004). Lower CSF Aβ1-42 could indicate reduced clearance of Aβ1-42, which has been hypothesized to be a result of amyloid accumulation in the brain (Tarasoff-Conway et al., 2015). Therefore, TBI events may be causing acute increases in amyloid production. However, several studies did not find an association between TBI and CSF (Neselius et al., 2012; Alosco et al., 2018; Weiner et al., 2022) and cerebral (Hicks et al., 2022) Aβ. Following a TBI event, axonal injury results in the accumulation of amyloid precursor protein, leading to intra-axonal Aβ aggregation and potentially Aβ aggregation and plaque formation. For this reason, CSF Aβ has been suggested as a potential biomarker for TBI (Tsitsopoulos and Marklund, 2013). There may also be other biological mechanisms at play in this pathway; for example, hypoperfusion, vascular dysfunction, and ischemia post-TBI may all contribute to Aβ deposition (Ramos-Cejudo et al., 2018). We found that age, *APOE* ε4, and cognition were all significantly associated with CSF Aβ1-42. Furthermore, the prevalence of TBI did not significantly differ between men (2.5%) and women (1.9%), indicating that the biological pathway operates independently of sex; yet, it may be mediated by a genetic predisposition for AD via *APOE* ε4 pathways (Jellinger et al., 2001).

Physical inactivity and lower BMI were both significantly associated with higher levels of p-tau 181 in CSF. Evidence suggests that exercise plays a critical role in reducing the accumulation of tau pathology and may be most beneficial in the preclinical phases of AD (Brown et al., 2019). In a study of cognitively normal participants, higher levels of self-reported physical activity predicted lower levels of CSF p-tau (Baker et al., 2012). Other research has found similar results with physical activity measured via actigraphy, where more time spent in moderate physical activity was significantly associated with lower levels of CSF p-tau (Law et al., 2018). On the other hand, several studies found different results: either the results attenuated after adjusting for covariates (Liang et al., 2010) or a lack of an association was observed in cognitively normal participants (Stojanovic et al., 2020; Roccati et al., 2023), pre-symptomatic autosomal-dominant AD (ADAD) (Brown et al., 2017), or AD patients via a 16-week moderate–high intense physical activity intervention (Steen Jensen et al., 2016). In our study, we found physical inactivity was significantly associated with higher levels of CSF p-tau. This relationship was significant even when adjusting for age, sex, cognition, and presence of *APOE* ε4. There are several potential mechanisms to explain this relationship. Physical activity has been shown to elicit a number of positive impacts on the brain, including an increase in levels of growth factors such as brain-derived neurotrophic factor (BDNF), altered inflammation, neurogenesis, and increased energy supply (Chen and Nakagawa, 2023). The relationship between these improvements and tau phosphorylation is less clear but may be due in part to increased clearance or altered production pathways. In this regard, the normal role of tau phosphorylation at specific sites, which occurs through a number of different kinase pathways such as glycogen synthase kinase 3 (GSK3) and cyclin-dependent kinase 5 (CDK5), is neither fully understood nor are the alterations in function that occur in disease states. *APOE* ε4 is a mediator of numerous pathological processes related to AD risk. *APOE* increases tau hyperphosphorylation, yet *APOE* ε4 carriers and non-carriers show similar benefits to brain health as a result of engagement in physical activity (Pearce et al., 2022). Given physical activity engagement entails a suite of biological processes, it is likely that many of these molecular targets align with AD neuropathology and therefore require further investigation (de Frutos Lucas et al., 2023). We also found lower BMI was associated with higher levels of p-tau 181 in CSF, where the prevailing evidence seems to indicate an association (Mathys et al., 2017; Bos et al., 2019; Zhang et al., 2022) rather than a lack of association (Pegueroles et al., 2020; Sun et al., 2020). There is considerable literature concerning an age-related risk matrix (Vidoni et al., 2011; Besser et al., 2016; Gottesman et al., 2017; Müller et al., 2017; Bos et al., 2019), a so-called “obesity paradox”, where lower BMI in midlife is associated with decreased AD risk while lower BMI in later life is associated with an increased risk. This paradox pattern appears to continue into the preclinical AD stage as indicated by biomarkers, where lower BMI has been associated with higher levels of CSF p-tau 181 in midlife (Mathys et al., 2017), and later life obesity has been linked with lower levels of CSF p-tau (Zhang et al., 2022).

We found that having more education and a higher BMI were both significantly associated with higher THV. Systematic review and meta-analysis from 45 observational, cross-sectional, epidemiological studies have demonstrated a clear association between higher BMI and lower brain volume (Han et al., 2021). However, there is some longitudinal evidence that changes in body weight are not related to hippocampal volume in later-life participants (Giudici et al., 2019). In our EPAD participants (mean age 66 years at baseline), higher BMI was associated with higher THV, adjusting for age and cognition, which were both significant. Previous research has found that midlife obesity (BMI > 30) was associated with an increased rate of hippocampal atrophy and global brain atrophy in cognitively normal individuals (Debette et al., 2011); however, in patients with AD, a negative correlation was observed (Ho et al., 2011). Given these findings and ours, it may be that the relationship between BMI and neurodegeneration is particularly important in midlife, where a higher BMI is associated with lower brain volume. This relationship is less important, however, in later life when changes in body weight do not seem to impact longitudinal changes in hippocampal volume.

In this study, we have shown how several key modifiable risk factors are associated with the AD hallmark AT(N) biomarkers. This study has substantial implications in clinical settings, where routine screening tests could use modifiable dementia risk factor profiles to assess risk, severity, and potential therapeutic interventions. Given modifiable risk factors often cluster together, addressing them in designated memory clinics could be a one-stop-shop for lifestyle modification, where we know precision medicine has the potential to catalyze positive behavior change (Freling et al., 2020). There are several strengths of our study of note. To the best of our knowledge, this is the first comprehensive investigation of modifiable dementia risk factors and AT(N) biomarkers. We used an open-source data repository from a large-scale, highly characterized, epidemiological cohort study, and our data and code are freely accessible and available to be reproduced. There are also several limitations. This was an observational study on existing longitudinal epidemiological data, and as such, no causal relationship can be construed. All participants were cognitively healthy at baseline; therefore, we were unable to include clinical staging on AT(N) instead of opting to adjust for cognition (MMSE) and age as proxies. Most self-reported measures were dichotomous, and hearing loss was omitted due to insufficient values for GAM comparison. Furthermore, the self-reported nature of medical history and risk factor adherence may have imparted bias to null, especially for participants who have experienced a TBI (McKinlay et al., 2016), where we were unable to account for injury severity. However, for the application of biomarker and risk factor interpretation in designated memory clinics or routine clinical practice, self-reported measures are commonplace and concordant with objective measures of risk. We were also unable to account for racial status as, in the EPAD LCS cohort, the participants were predominantly white Caucasian of European descent and not necessarily representative of the general population, which may limit the generalizability of our findings to other racial groups. Cognition was accounted for in our GAMs by adjusted for MMSE scores, which is a brief cognitive screening tool and not necessarily sensitive to precisely detecting early cognitive decline and individuals at risk (Gallegos et al., 2022). Finally, we acknowledge how modifiable risk factors have a tendency to cluster (Peters et al., 2019); however, this was outside the scope of our investigation. There is growing evidence that individual risk factors tend to interact, potentially leading to an underestimation of population attributable fraction (Welberry et al., 2023). Further research is necessary to elucidate the role of clustering risk factors and their impact on AT(N) biomarkers.

In conclusion, this study found that TBI, physical inactivity, lower BMI, and lower education were all significantly associated with increased burden of individual AT(N) biomarkers. Lifestyle modification offers an accessible, cost-effective, non-invasive, and easily targeted avenue for risk reduction. Given the strong evidence for modifiable risk factors being associated with AD incidence and prevalence, here we have shown significant relationships between several key modifiable dementia risk factors and the hallmark biomarkers of AD.

## Data availability statement

Publicly available datasets were analyzed in this study. This data can be found at: All EPAD data is available via the NTKApp on the AD Workbench, designed by ADDI. NTKApp is accessible at: https://www.alzheimersdata.org/ntk. R code is available upon request to the corresponding author, ER (eddy.roccati@utas.edu.au).

## Ethics statement

The studies involving humans were approved by the ethics committees of all participating EPAD centers. All procedures were conducted in accordance with the Declaration of Helsinki. The studies were conducted in accordance with the local legislation and institutional requirements. The participants provided their written informed consent to participate in this study.

## Author contributions

ER: Conceptualization, Formal analysis, Investigation, Methodology, Resources, Visualization, Writing – original draft, Writing – review & editing. AB: Conceptualization, Formal analysis, Investigation, Methodology, Visualization, Writing – original draft, Writing – review & editing. JC: Formal analysis, Investigation, Writing – review & editing. JE: Formal analysis, Investigation, Writing – review & editing. JB: Formal analysis, Investigation, Writing – review & editing. JA: Writing – review & editing. AK: Writing – review & editing. JV: Supervision, Writing – review & editing. MC: Writing – review & editing. CL: Writing – review & editing.
