# Supplementary material for: Modifiable dementia risk factors and AT(N) biomarkers: findings from the EPAD cohort
Source: Front Aging Neurosci. 2024 Feb 7;16:1346214. doi: 10.3389/fnagi.2024.1346214 (PMC10879413; doi:10.3389/fnagi.2024.1346214)
Supplement: Supplementary file 1 [file Data_Sheet_1.docx]

Supplementary Material

# Supplementary Tables

|  | | **A-** | **A+** | **Total** | **p-value** |
| --- | --- | --- | --- | --- | --- |
| **N (%)** | | 810 (66.6%) | 406 (33.4%) | 1216 |  |
| **Age at baseline in years (SD)** | | 65.2 (7.2) | 67.0 (7.2) | 65.9 (7.3) | <0.001 |
| **Education in years (SD)** | | 14.1 (3.7) | 14.5 (3.6) | 14.2 (3.9) | 0.311 |
| **MMSE (SD)** | | 28.7 (1.5) | 27.9 (2.4) | 28.4 (1.9) | <0.001 |
| ***APOE* ε4 genotype** | | | | | <0.001 |
|  | e2e2 | 3 (0.4%) | 0 (0%) | 3 (0.3%) |  |
|  | e2e3 | 81 (10.2%) | 21 (5.3%) | 102 (8.6%) |  |
|  | e3e3 | 472 (59.5%) | 151 (38%) | 623 (52.4%) |  |
|  | e2e4 | 21 (2.6%) | 9 (2.3%) | 30 (2.5%) |  |
|  | e3e4 | 207 (26.1%) | 174 (43.8%) | 381 (32%) |  |
|  | e4e4 | 9 (1.1%) | 42 (10.6%) | 51 (4.3%) |  |
| **A: CSF Aβ1-42 pg/mL (SD)** |  | 1723.8 (694.9) | 706.4 (188.9) | 1384.1 (750.9) | <0.001 |
| **T: CSF p-tau 181 pg/mL (SD)** |  | 18.6 (7.9) | 22.8 (15) | 20.0 (11) | <0.001 |
| **N: THV mm^3^ (SD)** |  | 4836.4 (573.8) | 4700.3 (646.4) | 4782.4 (607.1) | <0.001 |
| **Less education** | | | | | 0.175 |
|  | No | 542 (66.9%) | 255 (62.8%) | 797 (65.5%) |  |
|  | Yes | 268 (33.1%) | 151 (37.2%) | 419 (34.5%) |  |
| **Self-report hearing difficulty** | | | | | 1 |
|  | No | 28 (93.3%) | 11 (91.7%) | 39 (92.9%) |  |
|  | Yes | 2 (6.7%) | 1 (8.3%) | 3 (7.1%) |  |
| **TBI** | | | | | 0.923 |
|  | No | 737 (98.1%) | 364 (97.8%) | 1101 (98%) |  |
|  | Yes | 14 (1.9%) | 8 (2.2%) | 22 (2%) |  |
| **Hypertension** | | | | | 0.612 |
|  | No | 704 (93.7%) | 345 (92.7%) | 1049 (93.4%) |  |
|  | Yes | 47 (6.3%) | 27 (7.3%) | 74 (6.6%) |  |
| **Alcohol >21 units p/w** |  |  |  |  | 0.044 |
|  | No | 717 (89.4%) | 340 (85.2%) | 1057 (88%) |  |
|  | Yes | 85 (10.6%) | 59 (14.8%) | 144 (12%) |  |
| **Smoking** | | | | | 0.993 |
|  | No | 368 (45.6%) | 182 (45.4%) | 550 (45.5%) |  |
|  | Yes | 439 (54.4%) | 219 (54.6%) | 658 (54.5%) |  |
| **Obesity** | | | | | 0.013 |
|  | No | 276 (36.8%) | 166 (44.6%) | 442 (39.4%) |  |
|  | Yes | 475 (63.2%) | 206 (55.4%) | 681 (60.6%) |  |
| **Depression** | | | | | 0.188 |
|  | No | 705 (93.9%) | 357 (96%) | 1062 (94.6%) |  |
|  | Yes | 46 (6.1%) | 15 (4%) | 61 (5.4%) |  |
| **Physical inactivity** | | | | | 0.246 |
|  | No | 625 (77.4%) | 323 (80.5%) | 948 (78.5%) |  |
|  | Yes | 182 (22.6%) | 78 (19.5%) | 260 (21.5%) |  |
| **Diabetes** | | | | | 1 |
|  | No | 738 (98.3%) | 366 (98.4%) | 1104 (98.3%) |  |
|  | Yes | 13 (1.7%) | 6 (1.6%) | 19 (1.7%) |  |

**Supplementary Table 1.** **Summary statistics of included EPAD participants (N = 1216), stratified by amyloid classification [A+/-]**
All available data are displayed. All values shown are n (%) unless otherwise stated. Amyloid +/- was classified using a cut-off of CSF Aβ1-42: < 1000 pg/mL classified as A+ and > 1000 pg/mL classified as A-. All available data are displayed. P-values are provided for group comparisons (A+ v A-) between either continuous (One-way ANOVA) or categorical (Chi-squared) data. Significance was set at p < 0.05. SD = standard deviation; MMSE = Mini Mental State Examination; *APOE* = apolipoprotein epsilon E; Aβ = beta-amyloid; p-tau = phosphorylated tau; CSF = cerebrospinal fluid; THV = total hippocampal volume; TBI = traumatic brain injury.

|  | | **T-** | **T+** | **Total** | **p-value** |
| --- | --- | --- | --- | --- | --- |
| **N (%)** | | 1001 (82.4%) | 214 (17.6%) | 1215 |  |
| **Age at baseline in years (SD)** | | 65.0 (7.2) | 69.7 (6.3) | 65.9 (7.3) | <0.001 |
| **Education in years (SD)** | | 14.1 (3.7) | 14.6 (3.6) | 13.5 (4) | <0.001 |
| **MMSE (SD)** | | 28.7 (1.5) | 27.2 (2.8) | 28.4 (1.9) | <0.001 |
| ***APOE* ε4 genotype** | | | | | <0.001 |
|  | e2e2 | 3 (0.3%) | 0 (0%) | 3 (0.3%) |  |
|  | e2e3 | 90 (9.2%) | 12 (5.7%) | 102 (8.6%) |  |
|  | e3e3 | 542 (55.4%) | 80 (37.9%) | 622 (52.3%) |  |
|  | e2e4 | 26 (2.7%) | 4 (1.9%) | 30 (2.5%) |  |
|  | e3e4 | 290 (29.7%) | 91 (43.1%) | 381 (32%) |  |
|  | e4e4 | 27 (2.8%) | 24 (11.4%) | 51 (4.3%) |  |
| **A: CSF Aβ1-42 pg/mL (SD)** |  | 1393.9 (608) | 1342.2 (1214.6) | 1384.1 (750.9) | 0.360 |
| **T: CSF p-tau 181 pg/mL (SD)** |  | 16 (4.6) | 38.5 (13) | 20.0 (11) | <0.001 |
| **N: THV mm^3^ (SD)** |  | 4839.4 (582.9) | 4615.7 (645.4) | 4782.4 (607.1) | <0.001 |
| **Less education** | | | | | <0.001 |
|  | No | 678 (67.7%) | 118 (55.1%) | 796 (65.5%) |  |
|  | Yes | 323 (32.3%) | 96 (44.9%) | 419 (34.5%) |  |
| **Self-report hearing difficulty** | | | | | 1 |
|  | No | 31 (93.9%) | 8 (88.9%) | 39 (92.9%) |  |
|  | Yes | 2 (6.1%) | 1 (11.1%) | 3 (7.1%) |  |
| **TBI** | | | | | 0.168 |
|  | No | 899 (97.7%) | 201 (99.5%) | 1100 (98%) |  |
|  | Yes | 21 (2.3%) | 1 (0.5%) | 22 (2%) |  |
| **Hypertension** | | | | | 0.669 |
|  | No | 862 (93.7%) | 187 (92.6%) | 1049 (93.5%) |  |
|  | Yes | 58 (6.3%) | 15 (7.4%) | 73 (6.5%) |  |
| **Alcohol >21 units p/w** |  |  |  |  | 0.924 |
|  | No | 872 (88%) | 185 (88.5%) | 1057 (88.1%) |  |
|  | Yes | 119 (12%) | 24 (11.5%) | 143 (11.9%) |  |
| **Smoking** | | | | | 0.019 |
|  | No | 469 (47.1%) | 80 (37.9%) | 549 (45.5%) |  |
|  | Yes | 527 (52.9%) | 131 (62.1%) | 658 (54.5%) |  |
| **Obesity** | | | | | 0.025 |
|  | No | 347 (37.7%) | 94 (46.5%) | 441 (39.3%) |  |
|  | Yes | 573 (62.3%) | 108 (53.5%) | 681 (60.7%) |  |
| **Depression** | | | | | 0.603 |
|  | No | 872 (94.8%) | 189 (93.6%) | 1061 (94.6%) |  |
|  | Yes | 48 (5.2%) | 13 (6.4%) | 61 (5.4%) |  |
| **Physical inactivity** | | | | | 0.251 |
|  | No | 789 (79.2%) | 159 (75.4%) | 948 (78.5%) |  |
|  | Yes | 207 (20.8%) | 52 (24.6%) | 259 (21.5%) |  |
| **Diabetes** | | | | | 0.516 |
|  | No | 906 (98.5%) | 197 (97.5%) | 1103 (98.3%) |  |
|  | Yes | 14 (1.5%) | 5 (2.5%) | 19 (1.7%) |  |

**Supplementary Table 2.** **Summary statistics of included EPAD participants (N = 1215), stratified by tau classification [T+/-]**
All available data are displayed. All values shown are n (%) unless otherwise stated. Tau +/- was classified using a cut-off of CSF p-tau 181: > 27 pg/mL classified as T+ and < 27 pg/mL classified as T-. All available data are displayed. P-values are provided for group comparisons (T+ v T-) between either continuous (One-way ANOVA) or categorical (Chi-squared) data. Significance was set at p < 0.05. SD = standard deviation; MMSE = Mini Mental State Examination; *APOE* = apolipoprotein epsilon E; Aβ = beta-amyloid; p-tau = phosphorylated tau; CSF = cerebrospinal fluid; THV = total hippocampal volume; TBI = traumatic brain injury.

|  | | **N-** | **N+** | **Total** | **p-value** |
| --- | --- | --- | --- | --- | --- |
| **N (%)** | | 1105 (85.0%) | 195 (15.0%) | 1300 |  |
| **Age at baseline in years (SD)** | | 65.4 (7.1) | 68.7 (8) | 65.9 (7.3) | <0.001 |
| **Education in years (SD)** | | 14.1 (3.7) | 14.4 (3.7) | 14.1 (3.7) | 0.229 |
| **MMSE (SD)** | | 28.6 (1.6) | 27.6 (2.8) | 28.4 (1.9) | <0.001 |
| ***APOE* ε4 genotype** | | | | | 0.714 |
|  | e2e2 | 3 (0.3%) | 0 (0%) | 3 (0.2%) |  |
|  | e2e3 | 93 (8.8%) | 15 (8.1%) | 108 (8.7%) |  |
|  | e3e3 | 570 (53.7%) | 94 (50.8%) | 664 (53.2%) |  |
|  | e2e4 | 25 (2.4%) | 5 (2.7%) | 30 (2.4%) |  |
|  | e3e4 | 327 (30.8%) | 59 (31.9%) | 386 (31%) |  |
|  | e4e4 | 44 (4.1%) | 12 (6.5%) | 56 (4.5%) |  |
| **A: CSF Aβ1-42 pg/mL (SD)** |  | 1421.2 (754) | 1201.7 (713.1) | 1384.1 (750.9) | <0.001 |
| **T: CSF p-tau 181 pg/mL (SD)** |  | 19.6 (10.6) | 21.1 (11.9) | 20.0 (11) | 0.088 |
| **N: THV mm^3^ (SD)** |  | 4826.4 (579.1) | 4578.0 (688.2) | 4782.4 (607.1) | <0.001 |
| **Less education** | | | | | 0.864 |
|  | No | 723 (65.5%) | 126 (64.6%) | 849 (65.4%) |  |
|  | Yes | 380 (34.5%) | 69 (35.4%) | 449 (34.6%) |  |
| **Self-report hearing difficulty** | | | | | 1 |
|  | No | 31 (91.2%) | 8 (88.9%) | 39 (90.7%) |  |
|  | Yes | 3 (8.8%) | 1 (11.1%) | 4 (9.3%) |  |
| **TBI** | | | | | 0.547 |
|  | No | 993 (98.2%) | 175 (97.2%) | 1168 (98.1%) |  |
|  | Yes | 18 (1.8%) | 5 (2.8%) | 23 (1.9%) |  |
| **Hypertension** | | | | | 0.097 |
|  | No | 952 (94.2%) | 163 (90.6%) | 1115 (93.6%) |  |
|  | Yes | 59 (5.8%) | 17 (9.4%) | 76 (6.4%) |  |
| **Alcohol >21 units p/w** |  |  |  |  | 0.276 |
|  | No | 937 (87.8%) | 170 (90.9%) | 1107 (88.3%) |  |
|  | Yes | 130 (12.2%) | 17 (9.1%) | 147 (11.7%) |  |
| **Smoking** | | | | | 0.022 |
|  | No | 517 (47.5%) | 73 (38.2%) | 590 (46.1%) |  |
|  | Yes | 571 (52.5%) | 118 (61.8%) | 689 (53.9%) |  |
| **Obesity** | | | | | 0.021 |
|  | No | 382 (37.8%) | 85 (47.2%) | 467 (39.2%) |  |
|  | Yes | 629 (62.2%) | 95 (52.8%) | 724 (60.8%) |  |
| **Depression** | | | | | 0.568 |
|  | No | 952 (94.2%) | 172 (95.6%) | 1124 (94.4%) |  |
|  | Yes | 59 (5.8%) | 8 (4.4%) | 67 (5.6%) |  |
| **Physical inactivity** | | | | | 0.739 |
|  | No | 850 (78.2%) | 152 (79.6%) | 1002 (78.4%) |  |
|  | Yes | 237 (21.8%) | 39 (20.4%) | 276 (21.6%) |  |
| **Diabetes** | | | | | 0.293 |
|  | No | 997 (98.6%) | 175 (97.2%) | 1172 (98.4%) |  |
|  | Yes | 14 (1.4%) | 5 (2.8%) | 19 (1.6%) |  |

**Supplementary Table 3.** **Summary statistics of included EPAD participants (N = 1300), stratified by neurodegeneration classification [N+/-]**
All available data are displayed. All values shown are n (%) unless otherwise stated. Neurodegeneration +/- was classified using participants age and MTA average: participants were N+ if their age was <65 years and their MTA average (L/R) was > 1 or their age was > 65 years and their MTA average (L/R) was > 1.5. All available data are displayed. P-values are provided for group comparisons (N+ v N-) between either continuous (One-way ANOVA) or categorical (Chi-squared) data. Significance was set at p < 0.05. SD = standard deviation; MMSE = Mini Mental State Examination; *APOE* = apolipoprotein epsilon E; Aβ = beta-amyloid; p-tau = phosphorylated tau; CSF = cerebrospinal fluid; THV = total hippocampal volume; TBI = traumatic brain injury.
